# Percutaneous nephrolithotomy for management neglected encrusted ureteral stent in a transplanted kidney: a case report

**DOI:** 10.11604/pamj.2023.44.1.37682

**Published:** 2023-01-03

**Authors:** Abdul Azis, Syarif Bakri, Muh Zulharyahya Dandy Asmara Putra, Indrawarman Soehardjo

**Affiliations:** 1 Department of Surgery, Urology Division, Faculty of Medicine Hasanuddin University, Makassar, Indonesia,; 2 Department of Urology, Siloam Hospital, Makassar, Indonesia,; 3 Department of Surgery, Urology Division, Faculty of Medicine, Public Health and Nursing, Gadjah Mada University, Jogjakarta, Indonesia

**Keywords:** Urolithiasis, neglected DJ stent, transplanted kidney, percutaneous nephrolithotomy, case report

## Abstract

Retrieving these forgotten encrusted double j (DJ) stents could be challenging and require multimodal urological interventions, especially in a single-functioning transplanted kidney. Only a few cases of mini-percutaneous nephrolithotomy (mini-PCNL) via ultrasonography (US) guidance in transplanted kidneys have been published. We report a 47-year-old man; a case of a transplanted kidney, with a complaint of abdominal pain and dysuria for two weeks. The patient had a forgotten DJ stent for more than a year due to a lack of post-procedural follow-up information. An abdominal computed tomography scan showed a 20 x 13mm stone in the proximal tip of the DJ stent. Ultrasonography-guided mini-PCNL with a 19-fr rigid nephroscope was performed without complications. In conclusion, we emphasize the importance of patient education regarding the indwelling DJ stent. This case also provides that if an experienced urologist performs it, the US-guided mini-PCNL is safe and effective in transplanted kidney patients.

## Introduction

Urolithiasis is a rare complication among kidney transplant recipients, with a prevalence rate of 0.23-6.3% [1]. This condition can cause a urinary obstruction that leads to an increase in morbidity and a decrease in kidney function [1]. Today, a DJ stent has become the most comprehensive device in urological procedures. The benefits of DJ stent placement in the construction of a ureteroneocystostomy are to reduce urinary leaks and avoid obstruction in the early post-trans-plant period [2]. Forgotten encrusted double j (DJ) stents are a well-recognized phenomenon with the potential to cause many complications, especially in a single-functioning transplanted kidney [3]. Retrieving these ureteric stents could be challenging and may require multimodal urological interventions, such as extracorporeal shock wave lithotripsy (ESWL), ureteroscopy (URS), percutaneous nephrolithotomy (PCNL), and open surgery [1]. The changing position of the transplanted kidney from its actual position to the adjusted position in the recipient´s abdomen. This adjusted position results in not all procedures can be performed [4,5]. Only a few cases of mini-percutaneous nephrolithotomy (mini-PCNL) via ultrasonography (US) guidance in transplanted kidneys have been published [1,4]. Here, we report our experience handling forgotten encrusted DJ stent in transplanted kidney patient using a mini PCNL procedure with US guidance.

## Patient and observation

**Patient information:** a 47-year-old man with a right iliac fossa allograft renal transplantation history came to a urology specialist complaining of lower right abdominal pain and dysuria for two weeks. The patient has a history of chronic kidney disease and has undergone hemodialysis since 2017. Furthermore, he underwent renal allograft transplantation in 2019. Unfortunately, the patient was lost to transplant clinic follow-up after the renal transplantation procedure. The patient has a history of hypertension and had a non-hemorrhagic stroke three months ago.

**Clinical findings:** the physical examination showed mild tenderness in the right lower quadrant side of the abdomen.

**Figure 1:**
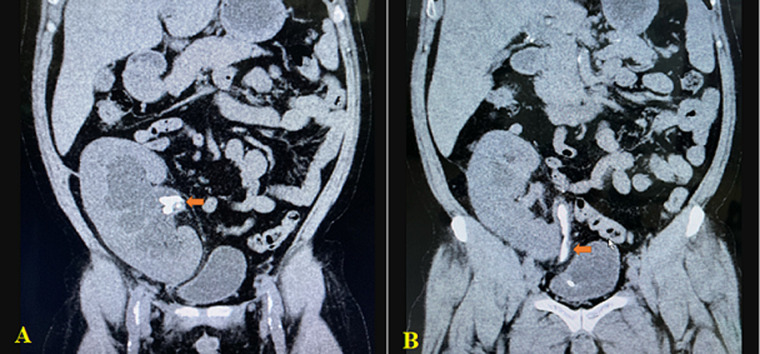
coronal computed tomography image showing stones in A) the pelvis of the transplanted kidney (arrow); B) the distal DJ stent (arrow)

**Diagnostic assessment:** the patient's serum creatinine and blood urea nitrogen (BUN) concentrations were within normal limits at a value of 1.10 mg/dL and 37 mg/dL. A non-contrast abdomen pelvic computed tomography (CT) scan showed moderate hydronephrosis of the transplanted kidney and stone formation in the proximal tip of the DJ stent measuring 20 x 13mm, total encrusted DJ stent (Figure 1A), and stone formation in the lower DJ stent (Figure 1B).

**Figure 2:**
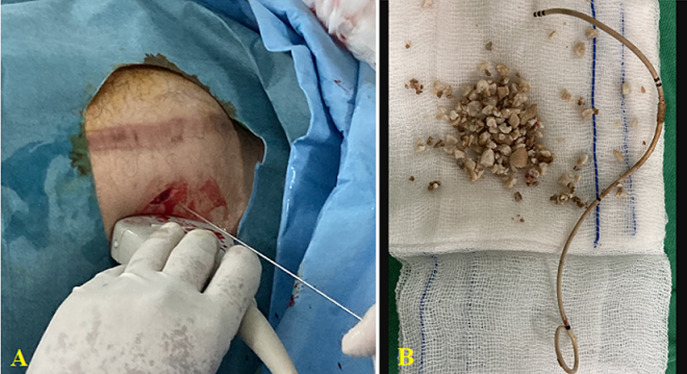
A) percutaneous punctures to the kidney with ultrasound guide; B) stones and DJ stents were successfully removed

**Therapeutic intervention:** first, preoperative preparation was performed by administration of ceftriaxone 1g intravenously 2 hours before the surgery. After receiving general anesthesia, the patient was positioned in the lithotomy position. Initially, the patient was planned to undergo a conventional PCNL process with fluoroscopy guidance. However, due to DJ stent encrustation and stone formation, the removal of DJ stent and replacement of a ureteral catheter cannot be performed. For that, the urologist decided to perform mini-PCNL with US guidance. We first performed a cystolitholapexy to remove a bladder stone on the lower tip of DJ stent. Then, with supine position, a color Doppler US with 3.5 MHz probe (BK Medical) was used for pelvicalyceal system visualization. Next, an incision was made in the skin, and an 18g needle was inserted using a US guide to access the suitable calyx of the kidney (Figure 2A). After that, a 0.035-inch j tipped guide wire was inserted into the aimed calyx. Metal dilators dilate the nephrostomy tract starting with sizes 10, 12, 15, and 20 Fr. Then, the dilators were removed, leaving the amplatz sheath and guide wire in place. Next, a 19 fr rigid nephroscope was inserted to the pyelocaliceal system, and a warm saline solution was used for irrigation. The stone was identified and fragmented by lithoclast and extracted with forceps without residue (Figure 2B). Stone-free status was rechecked at the end of the procedure using US, and then a new DJ stent was installed. The total operative time was about 115 minutes and the estimated blood loss was 100ml.

**Follow-up and outcomes:** the patient's post-surgical recovery was unremarkable. A kidney, ureter, and bladder (KUB) X-ray was performed at the second postoperative day, and the results showed a DJ stent was installed in a good position without residual stone. The urethral foley was removed after 24 hours. The patient was discharged with oral antibiotics on the third postoperative day. The DJ stent was removed four weeks later.

**Patient´s perspective:** the patient was satisfied with the successful outcome of the surgery.

**Informed consent:** written informed consent was obtained from the patient for participation in our study.

## Discussion

Stone formation in kidney transplant recipients is sporadic, only around 0.4%-1% compare to other kidney transplant complications [5]. Secondary hyperparathyroidism, urinary tract infections, and renal tubular acidosis are the main risk factors for stone formation in these patients. While outflow obstruction, foreign bodies (e.g., stents, sutures), and donor lithiasis are factors that rarely cause stones formation in kidney transplant patients [5,6]. Double j stent placement is a standard procedure performed by many urologists, especially after performing a multitude of reconstructive surgeries, such as renal transplantation, to keep the ureter patent, ensure resolution of any edema, allow any injury to heal, reduce urinary leak, and avoiding obstruction in the early post-transplant period [7]. Long-term DJ stents can lead to encrustations, stone formation, fractures and blockades of stents, hydronephrosis, infections, and subsequently loss of renal function [8]. The encrustation can occur in both infected and sterile urine, and the exact mechanism resulting in encrustation is unknown. The extent of the encrustation is closely linked to the length of time the DJ stent remains in situ [9]. Kawahara *et al*. reported an encrustation rate of 26.8% if less than six weeks, 56.9% at 6 to 12 weeks, and 75.9% after 12 weeks from insertion [10]. In our case, the patient had a DJ stent for more than one year and was totally encrusted. Poor patient compliance is the most common reason for long-standing DJ stents, which can be explained to some extent by the patients' low educational level. Other reasons are ignorance of the DJ stent and inability to access the hospital (poverty) [11]. In our case, the lack of post-procedural follow-up information was the reason for long-standing DJ stent. Patil *et al*. report that most of the patients presenting with forgotten DJ stent were from poor socioeconomic backgrounds and had low education status [8].

There are several ways to avoid missing DJ stents, including computerized monitoring programs, stent removal software, and follow-up via e-mail, phone, and mobile text messages. These techniques may reduce the likelihood of stent neglect by physicians and patients [3]. Several modalities can be used for nephrolithiasis in transplanted kidneys. While the choice is based on the stone size, position of the transplanted kidney, and surgeon´s experiences. In the case of small stones (0.5-1.5 cm), extracorporeal shock wave lithotripsy( ESWL) can be used as a safe modality [4]. However, because the kidney is located over the bony pelvis, identifying such stones can be difficult. Furthermore, due to the difficulty in identifying the ureteric orifice, stein strasse if happened, it may, can be problematic [12]. In some reports, URS has also been used successfully on small stones. However, using this modality is not easy because of the difficulties in accessing the ureteral orifice of the transplanted kidney, and there is a risk of injury [1,12]. In large stones with more than 2 cm, the ideal modality is PCNL, especially in transplanted kidneys whose anatomical location is in the iliac fossa, and the kidney surface is close to the skin [4,5]. Mini-PCNL is performed with small percutaneous tract sheaths Fr 11-20. In addition, when compared to conventional PCNL, mini-PCNL has less blood loss, increased intrarenal flexibility, significantly reduced postoperative pain, and a shorter hospital stay. One limitation is the need to disintegrate stones into small enough fragments to fit through a smaller sheath, resulting in longer operative times [1]. In a previous paper on managing kidney stones in transplanted kidneys, Ali Eslahi *et al*. reported their experiences with the mini-PCNL procedure via US guidance in two transplanted cases without any complications [1]. In our case, the DJ stent was totally encrusted and conventional PCNL with fluoroscopy guidance was difficult to perform. For that, we used US-guided mini-PCNL. The advantages of US-guided mini-PCNL include continuous monitoring of the deeper structures and vessels during the operation, accurate estimations of access to the stone, no radiation exposure for the staff, and no need for contrast injection [13].

## Conclusion

To prevent cases of forgotten or neglected DJ stents in the future, urologists need to pay more attention to informing and educating patients on post-operative procedures and follow-up. This case provides that if an experienced urologist performs it, the US-guided mini-PCNL is safe and effective in transplanted kidney patients.
